# Salinity-Induced Modulation of Phenolic Compounds and Antioxidant Activity in *Lavandula viridis* and *Thymus lotocephalus*

**DOI:** 10.3390/biom16081178

**Published:** 2026-08-12

**Authors:** Inês Mansinhos, Sandra Gonçalves, Raquel Rodríguez-Solana, Gema Pereira-Caro, Anabela Romano

**Affiliations:** 1 MED-Mediterranean Institute for Agriculture, Environment and Development & CHANGE-Global Change and Sustainability Institute, Faculdade de Ciências e Tecnologia, Universidade do Algarve, Campus de Gambelas, 8005-139 Faro, Portugal; ifmansinhos@ualg.pt (I.M.); raquel.rodriguez.solana@juntadeandalucia.es (R.R.-S.); 2 Department of Agroindustry and Food Quality, Andalusian Institute of Agricultural and Fisheries Research and Training (IFAPA), Rancho de la Merced Center, Carretera Cañada de la Loba (CA-3102) Km 3.1., SN, 11471 Jerez de la Frontera, Cádiz, Spain; 3 Department of Agroindustry and Food Quality, Andalusian Institute of Agricultural and Fisheries Research and Training (IFAPA), Alameda del Obispo Center, Avenida Menendez-Pidal, SN, 14004 Córdoba, Córdoba, Spain; mariag.pereira@juntadeandalucia.es; 4 Foods for Health Group, Instituto Maimónides de Investigación Biomédica de Córdoba (IMIBIC), Avenida Menendez-Pidal, SN, 14004 Córdoba, Córdoba, Spain

**Keywords:** abiotic stress, antioxidant activity, UHPLC-HRMS-MS, in vitro culture, Lamiaceae, Mediterranean plants, phenolic compounds, salinity, elicitation

## Abstract

Mediterranean Lamiaceae species are important sources of bioactive compounds with potential applications in food, pharmaceutical, and cosmetic industries. In the present study, controlled salinity was evaluated as a biotechnological elicitation strategy to enhance the accumulation of bioactive metabolites in two species, *Lavandula viridis* and *Thymus lotocephalus*, cultured in vitro. Morphological, biochemical and metabolic responses of shoots grown for seven weeks in culture medium containing four NaCl concentrations (0, 25, 50, or 75 mM) were assessed. Mild salinity (25 mM NaCl) stimulated shoot growth in both species, whereas higher salt concentrations progressively reduced growth. Salinity induced oxidative stress, as indicated by elevated malondialdehyde (MDA) levels at 25 mM NaCl in both species, while hydrogen peroxide (H_2_O_2_) exhibited contrasting patterns, decreasing with increasing salinity in *L. viridis* but peaking at 50 mM NaCl in *T. lotocephalus*. Salt stress caused reductions in photosynthetic pigments, phenolic compounds, antioxidant capacity (DPPH, FRAP, ABTS, and ORAC), and overall metabolic performance in *L. viridis*, indicating limited tolerance to salinity. Conversely, *T. lotocephalus* displayed greater metabolic plasticity, characterized by increased levels of phenolic compounds, particularly rosmarinic acid and its derivatives, and higher antioxidant activity of the extracts, as well as the accumulation of proline and soluble sugars. These findings demonstrate marked species-specific differences in salinity responses and identify controlled salinity as an effective elicitation strategy to sustainably produce high-value bioactive compounds in *T. lotocephalus*.

## 1. Introduction

Climate change is one of the most pressing environmental challenges of the 21st century, posing significant threats to plant biodiversity and ecosystem resilience [1]. Rising global temperatures, erratic precipitation patterns, increasing soil salinity, and more frequent extreme weather events expose plants to multiple abiotic stresses that adversely affect growth, development, and survival [2]. These environmental constraints disrupt key physiological and metabolic processes, including water uptake, nutrient acquisition, and photosynthesis, ultimately reducing plant productivity, destabilizing crop yields, and accelerating biodiversity loss [3]. Endemic and rare species are particularly vulnerable, as their restricted geographic distribution and limited adaptive capacity increase their extinction risk under rapidly changing environmental conditions [4]. 

Among abiotic stresses, soil salinity is a primary factor limiting agricultural productivity worldwide. It is estimated that salinization could affect more than 50% of arable land by 2050 [5]. Soil salinity results from the excessive accumulation of soluble salts, mainly Na^+^, Cl^‒^, SO_4_^2‒^, Mg^2+^ and Ca^2+^ [6]. It may originate from natural processes (primary salinization) prevalent in arid and semi-arid regions due to low rainfall, high evapotranspiration, and capillary rise of saline groundwater, or from anthropogenic activities (secondary salinization), such as irrigation with poor-quality water and excessive fertilizer application [5,7]. Furthermore, salinity can be classified as neutral (e.g., NaCl) or alkaline (e.g., Na_2_CO_3_ or K_2_CO_3_), the latter being associated with elevated soil pH that further aggravates plant and microbial stress [8].

Salinity alters soil physicochemical properties by reducing porosity, aeration, and hydraulic conductivity, thereby restricting water availability and nutrient uptake [9]. The resulting stress primarily involves two interconnected mechanisms: (i) osmotic stress, caused by reduced soil water potential, which limits water absorption, and (ii) ionic toxicity resulting from excessive accumulation of Na^+^ and Cl^−^, leading to membrane destabilization and cellular damage [7]. To cope with these constraints, plants undergo a range of morphological, anatomical, physiological, and biochemical adaptations. A primary response includes increasing root-to-shoot biomass ratio, which optimizes water acquisition while restricting the translocation of toxic ions to photosynthetically active tissues [10].

Excessive accumulation of Na^+^ and Cl^‒^ also promotes the overproduction of reactive oxygen species (ROS), triggering oxidative stress and lipid peroxidation [9,11]. Although severe oxidative stress is detrimental, moderate abiotic stress can act as an elicitor, stimulating the biosynthesis of secondary metabolites, including phenolic compounds and terpenoids, which function as non-enzymatic antioxidants to aid in ROS detoxification [6]. This stress-induced metabolic reprogramming has attracted considerable attention because many of these compounds possess antioxidant, anti-inflammatory, antimicrobial, and other bioactive properties of high value for the pharmaceutical, cosmetic, and food industries [6].

Plant tissue culture provides a powerful platform for investigating plant responses to abiotic stress while offering a sustainable approach for producing high-value secondary metabolites. Compared with conventional cultivation, in vitro systems enable the production of bioactive compounds under controlled and aseptic conditions, independent of seasonal or geographical constraints, and reduces harvesting pressure on wild populations of threatened species [12,13]. Moreover, these systems allow the application of elicitation strategies and metabolic engineering approaches to enhance or even induce the biosynthesis of target metabolites that are absent or present only in trace amounts in naturally grown plants [13].

The Lamiaceae is one of the most economically significant families of medicinal and aromatic plants, renowned for producing essential oils and phenolic compounds with widespread industrial applications [6]. Many Mediterranean Lamiaceae species are particularly vulnerable to climate change due to their ecological specialization and restricted distribution [14]. Among them, *Lavandula viridis* L’Hér and *Thymus lotocephalus* G. López and R. Morales are endemic to the Iberian Peninsula and the Algarve region (southern Portugal), respectively. Both species are rich sources of rosmarinic acid (RA), and previous studies have demonstrated that their in vitro cultures accumulate higher concentrations of this phenolic compound than micropropagated plants grown in ex vitro conditions [15]. Despite being relatively understudied, both species have attracted increasing scientific interest owing to their diverse biological properties, including the antioxidant, antimicrobial, antifungal, antiprotozoal, nematocidal, and inhibitory effects of enzymes associated with diabetes, neurodegenerative diseases, and skin ageing [12].

Against this background, understanding the mechanisms underlying plant responses to salinity is essential both for developing climate-resilient crops and for optimizing the production of valuable secondary metabolites through controlled elicitation. Therefore, this study aimed, for the first time, to evaluate the morphological and biochemical responses of *L. viridis* and *T. lotocephalus* in vitro shoots to salinity. In addition, the impact of salinity on the phenolic profile, determined by Ultra-High-Performance Liquid Chromatography coupled to High-Resolution Mass Spectrometry (UHPLC-HRMS-MS), and on the antioxidant activity of green-solvent extracts was comprehensively investigated.

## 2. Materials and Methods

### 2.1. Plant Material and Salinity Treatments

In vitro shoots of *L. viridis* and *T. lotocephalus*, established from multiple genotypes collected from natural populations and maintained under standard in vitro culture procedures according to Dias et al. [16] and Coelho et al. [17], respectively, were used in this study. Previously optimized multiplication media were used throughout the experiment for each species to ensure species-specific normal shoot development. Accordingly, *L. viridis* shoots were cultured on Murashige and Skoog (MS) medium [18] supplemented with 0.2 mg L^−1^ 6-benzyladenine (BA), whereas *T. lotocephalus* shoots were cultured on hormone-free MS medium. Media contained 2% (*w*/*v*) sucrose and 0.8% (*w*/*v*) agar. To evaluate the effects of salinity, sodium chloride (NaCl) was added to the basal medium at final concentrations of 25, 50, or 75 mM. These selected concentrations were based on the reported salinity sensitivity of Lamiaceae species and on previous studies indicating that salinity levels ranging from 25 to 100 mM are sufficient to induce physiological and biochemical stress responses while maintaining plant viability [19,20,21,22]. Higher concentrations (100 and 150 mM) were also tested in preliminary assays but proved highly phytotoxic and were therefore excluded from subsequent experiments. Shoots cultured on the corresponding multiplication media without NaCl served as the control treatment (CT). Culture media (80 mL) were dispensed into 250 mL Erlenmeyer flasks and sterilized at 121 °C for 20 min. Seven shoots were cultured per flask, and twelve flasks were tested for each treatment. Cultures were maintained at 25 ± 1 °C under a 16 h light/8 h dark photoperiod provided by cool-white fluorescent lamps (40 μmol m^−2^ s^−1^). After seven weeks of culture, morphological parameters were recorded. Plant material for biochemical analyses was immediately frozen in liquid nitrogen and stored until analysis, whereas material intended for extract preparation was oven-dried at 40 °C to constant weight.

### 2.2. Shoot Growth Evaluation

Seven shoots were initially inoculated per culture flask. After seven weeks of culture, four shoots were randomly selected from each flask for growth evaluation. The number of shoots produced by each selected shoot and the length of the longest shoot were recorded. The same four shoots were pooled for fresh weight determination, generating one fresh biomass value per flask. Dry weight was subsequently determined after oven-drying the pooled shoots at 40 °C until constant weight.

### 2.3. Determination of Chlorophyll and Carotenoid Contents

Photosynthetic pigments were quantified according to the method of Lichtenthaler [23]. Fresh plant material (25 mg) was homogenized in 4 mL of pure acetone and centrifuged. The absorbance of the supernatant was measured at 470, 644.8, and 661.6 nm using a T70+ UV/Vis spectrophotometer (PG Instruments Ltd., Leicestershire, UK). The contents of chlorophyll *a*, chlorophyll *b*, total chlorophyll, and carotenoids and the chlorophyll *a*/*b* and total chlorophyll/carotenoid ratios were calculated for each salinity treatment. The results were expressed as milligrams per gram of fresh weight (mg g^−1^_FW_). 

### 2.4. Determination of Hydrogen Peroxide and Lipid Peroxidation

Hydrogen peroxide (H_2_O_2_) and lipid peroxidation were determined according to Mansinhos et al. [24]. Approximately 100 mg of fresh plant material was homogenized in 1 mL of 0.1% (*w*/*v*) trichloroacetic acid (TCA) and centrifuged. The resulting supernatant was used for both analyses. For H_2_O_2_ determination, 0.2 mL of supernatant was mixed with 0.2 mL of 10 mM potassium phosphate buffer and 0.4 mL of 1 M potassium iodide, and absorbance was measured at 390 nm after 30 min using a Synergy^TM^ HTX MultiMode Microplate Reader (BioTek Instruments, Inc., Winooski, VT, USA). H_2_O_2_ concentration was determined from a calibration curve prepared with H_2_O_2_ standards and expressed as H_2_O_2_ equivalents per gram of fresh weight (µmol_H2O2_ g^−1^_FW_). Lipid peroxidation was estimated by measuring thiobarbituric acid-reactive substances (TBARSs) such as malondialdehyde (MDA). Briefly, 0.5 mL of supernatant was mixed with 0.5 mL of either a reaction solution containing 0.5% (*w*/*v*) TBA and 20% (*w*/*v*) TCA or a blank solution containing 20% (*w*/*v*) TCA. Samples were incubated at 95 °C for 30 min, rapidly cooled on ice, and absorbance was recorded at 440, 532, and 600 nm. MDA content was expressed as MDA equivalents per gram of fresh weight (nmol_MDA_ g^−1^_FW_).

### 2.5. Determination of Soluble Sugars and Proline

Soluble sugar content was determined according to Abid et al. [25]. Fresh plant material (25 mg) was homogenized in 2 mL of 80% (*v*/*v*) ethanol and incubated in a water bath at 80 °C for 30 min. After centrifugation, 100 μL of supernatant was mixed with an equal volume of distilled water, followed by the addition of 200 μL of 9% (*w*/*v*) phenol and 1 mL of concentrated sulfuric acid. After incubation for 30 min, absorbance was measured at 490 nm using the microplate reader. The results were expressed in milligrams of glucose equivalents per gram of fresh weight (mg_GLU_ g^−1^_FW_). Proline content was determined according to Mansinhos et al. [24]. Fresh plant material (25 mg) was subjected to three successive extractions with 0.5 mL of 80% (*v*/*v*) ethanol at 80 °C for 30 min each. The combined extracts were incubated with 1% (*w*/*v*) ninhydrin prepared in 60% (*v*/*v*) acetic acid for 1 h at 100 °C. After cooling, 1 mL of toluene was added, and the absorbance of the organic phase was measured at 520 nm. The results were expressed as micrograms of proline equivalents per gram of fresh weight (µg_PRO_ g^−1^_FW_).

### 2.6. Extraction, Profiling, and Antioxidant Capacity Evaluation of Phenolic Compounds

#### 2.6.1. Combined NADES and Ultrasound-Assisted Extraction (NADES-UAE)

Plant material was oven-dried at 40 °C to constant weight and ground to a particle size of <2 mm. Phenolic compounds were extracted using a natural deep eutectic solvent (NADES) composed of choline chloride and lactic acid (1:2 molar ratio) containing 30% (*w*/*w*) distilled water. This solvent was selected based on its demonstrated efficiency for extracting phenolic compounds from Lamiaceae species and the recognized advantages of NADESs as environmentally friendly extraction media [15,24,26]. Extractions were performed at a plant-to-solvent ratio of 2.5:100 (*w*/*v*) using an ultrasound-assisted extraction (UAE) bath (Elmasonic S 100 H, 220–240 V, 550 W; Elma Hans Schmidbauer GmbH & Co. KG, Singen, Germany). Samples were sonicated at a frequency of 37 kHz (sweep function) for 30 min at 50 °C. The extracts were subsequently filtered and stored at −20 °C until further analysis.

#### 2.6.2. Phenolic Profiling by HPLC-HRMS

The phenolic composition of the extracts was characterized by an HPLC-HRMS-MS system equipped with a Vanquish Neo UHPLC system (Thermo Scientific, San José, CA, USA) and coupled to an Orbitrap Exploris 240 high-resolution mass spectrometer (Thermo Scientific, San José, CA, USA). For chromatographic separation, a Hypersil GOLD column (100 × 1.0 mm i.d., 1.9 µ Thermo Scientific) was used and thermostated at 40 °C. The autosampler temperature was kept at 10 °C. The mobile phase consisted of 0.1% aqueous formic acid solution (solvent A) and can/deionized water (80, 20, *v*/*v*) acidified with 0.1% formic acid (solvent B) with the followed gradient programme: 0–2 min, 5% B; 2–18 min, 5–95%; 18–22 min, 95% B. Then, the column was re-equilibrated to the initial conditions; the flow rate was 0.05 mL/min. The Orbitrap Exploris 240 operated at a resolution of 180,000 and was fitted with a Heated Electrospray Ionization Probe (H-ESI) in negative ionization mode for the determination of secondary metabolites (from 70 to 1000 *m*/*z*). H-ESI parameters were as follows: −2.5 kV spray voltage, 20 arbitrary units (AU) of sheath gas (N_2_), 10 AU of auxiliary gas (N_2_), ion transfer tube temperature at 325 °C, and vaporizer temperature at 120° C. The instrument was operated in Full Scan and Data-Dependent MS2 modes.

Data acquisition and processing were performed using Xcalibur software (version 4.6, Thermo Fisher Scientific, San Jose, CA, USA). Quality control (QC) samples, prepared by pooling equal aliquots of representative extracts, were periodically injected throughout the analytical sequence to monitor instrumental stability and analytical reproducibility, maintaining metabolite variability below 20%. Compound identification was based on the analysis of authentic standards, considering retention times and accurate masses. When standards were unavailable, tentative identification was achieved by comparing the measured exact mass of the molecular ion with theoretical values and consulting metabolite databases, including PubChem, Phenol-Explorer, Metlin, and ChemSpider. Metabolite annotation followed the MSI reporting criteria proposed by Sumner et al. [27]. Information regarding molecular formulae, exact masses, mass accuracy (Δppm), retention times (RT), and MSI identification levels is provided in Table S1. Validation parameters used for phenolic quantification, including linearity ranges, slopes, intercepts, coefficients of determination (R^2^), limits of detection (LOD), and limits of quantification (LOQ), are presented in Table S2. LOD and LOQ values ranged from 0.02 to 49.61 µg L^−1^ and from 0.06 to 150.35 µg L^−1^, respectively. Quantitative results were expressed as µg g^−1^_DW_.

#### 2.6.3. Antioxidant Capacity Assessment

The antioxidant capacity of the green extracts was assessed using the procedure outlined by Mansinhos et al. [24], which combines four complementary assays. The ferric reducing antioxidant power (FRAP) assay is based on a single-electron transfer mechanism, whereas the oxygen radical absorbance capacity (ORAC) assay measures hydrogen atom transfer. The ABTS [2,2′-azino-bis(3-ethylbenzothiazoline-6-sulfonic acid)] and DPPH (2,2-diphenyl-1-picrylhydrazyl) assays involve both reaction mechanisms. The results are reported as milligrams of ascorbic acid equivalents per gram of dry weight (mg_AAE_ g^−1^_DW_) for the FRAP assay, and as milligrams of Trolox (6-hydroxy-2,5,7,8-tetramethylchroman-2-carboxylic acid) equivalents per gram of dry weight (mg_TE_ g^−1^_DW_) for the ABTS, DPPH, and ORAC assays.

### 2.7. Statistical Analysis

Data are presented as the mean ± standard error (SE). Growth parameters were obtained from five independent flasks per treatment (*n* = 5), with each flask considered an experimental unit. For each flask, the mean shoot number and mean shoot length were calculated from the four randomly selected shoots and used for statistical analyses. Fresh and dry biomass were determined from the pooled four selected shoots from each flask, generating one biomass value per flask. All biochemical analyses were performed using three independent biological replicates per treatment (*n* = 3). Each biological replicate consisted of plant material pooled from independent culture flasks within the same treatment. Prior to statistical analysis, the assumptions of normality and homogeneity of variances were assessed using the Shapiro–Wilk and Levene’s tests, respectively. Statistical differences were assessed through one-way analysis of variance (ANOVA) followed by Duncan’s new multiple range test (*p* < 0.05). For variables in which the assumption of homogeneity of variances was not met, Welch’s ANOVA was additionally performed to assess the robustness of the results. The robust analyses confirmed the same pattern of statistical significance as the conventional ANOVA. For multivariate analysis, OriginPro software (version 2024, OriginLab Corporation, Northampton, MA, USA) was employed using the mean values of each treatment. Variables were standardized using z-score normalization prior to heatmap and hierarchical cluster analysis (HCA). Compounds that were not detected or presented concentrations below the LOQ were excluded from the multivariate dataset. Hierarchical clustering was performed using Euclidean distance and the Group Average linkage method.

## 3. Results and Discussion

As each species was maintained on its own previously optimized multiplication medium, the following discussion focuses on species-specific responses to salinity. 

### 3.1. Growth Parameters 

**Figure 1:**
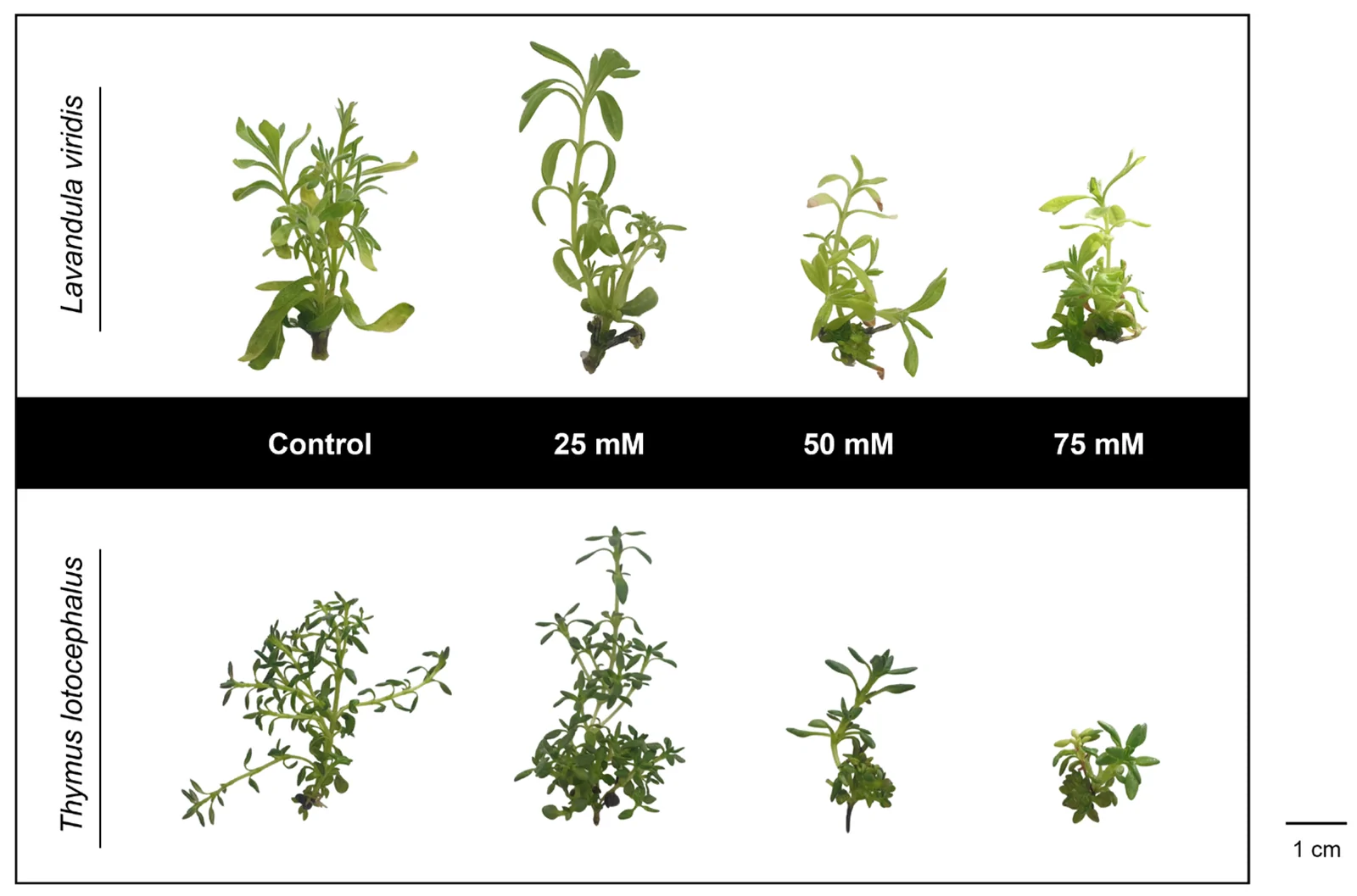
Shoots of *Lavandula viridis* and *Thymus lotocephalus* cultured on control medium (CT; 0 mM NaCl) and media with 25, 50, or 75 mM NaCl. The scale bar corresponds to 1 cm.

The effects of NaCl on the growth of *L. viridis* and *T. lotocephalus* shoots cultured under control conditions and in media containing 25, 50, or 75 mM NaCl are presented in Table 1 and Figure 1. 

In both species, increasing NaCl concentration generally reduced growth performance, a response widely reported in Lamiaceae species, in which salt stress adversely affects morphological, physiological, and biochemical processes [20,28]. Nevertheless, mild salinity (25 mM NaCl) was associated with slight improvements in specific growth traits, including increased length of the longest shoots in *L. viridis* and increased shoot number and fresh biomass in *T. lotocephalus*. Similar responses to moderate salinity have previously been described in related species such as *Salvia coccinea*, where low salt concentrations promoted plant growth [29].

The number of shoots, shoot length, fresh weight, and dry weight markedly declined in *L. viridis* at 50 and 75 mM NaCl, demonstrating the pronounced sensitivity of this species to salinity. This response agrees with observations in *Lavandula angustifolia*, in which NaCl concentrations above 50 mM significantly reduced plant growth and induced visible symptoms of stress, including chlorosis and necrosis [19]. In contrast, *Lavandula multifida* has been reported to tolerate NaCl levels of up to 60 mM without significant reductions in biomass accumulation [30]. The highest NaCl levels (50 and 75 mM) also significantly reduced shoot development and biomass production in *T. lotocephalus*. Similar growth inhibition has been reported for *Thymus vulgaris* and *Thymus daenensis*, in which 60 mM NaCl or higher levels markedly decreased dry matter accumulation [20]. Conversely, *Thymus pannonicus* exhibited only minor growth reductions under the same salinity level, highlighting the considerable interspecific variability in salt tolerance within the genus [20].

Growth inhibition is one of the earliest plant responses to salinity stress. The osmotic imbalance caused by elevated salt concentrations rapidly decreases cellular turgor, restricting cell expansion and shoot growth. As stress persists, plant metabolism is progressively reprogrammed, shifting resource allocation from growth and primary metabolism towards protective and defence mechanisms, including osmotic adjustment and antioxidant responses [1]. This inhibitory pattern aligns with our previous findings under polyethylene glycol (PEG)-induced osmotic stress, where increasing PEG concentrations progressively reduced *T. lotocephalus* shoot length [31]. Likewise, nutrient deprivation significantly impaired the growth of both *T. lotocephalus* and *L. viridis*, with nitrogen deficiency exerting the strongest inhibitory effect on shoot development and biomass accumulation [12].

Overall, these findings demonstrate that both species are sensitive to salinity stress, although *T. lotocephalus* exhibited a greater capacity to tolerate moderate salt concentrations. The responses observed at 25 mM NaCl suggest that mild salinity may act as an effective elicitor, whereas higher concentrations progressively compromise shoot growth and development.

### 3.2. Photosynthetic Pigments

**Figure 2:**
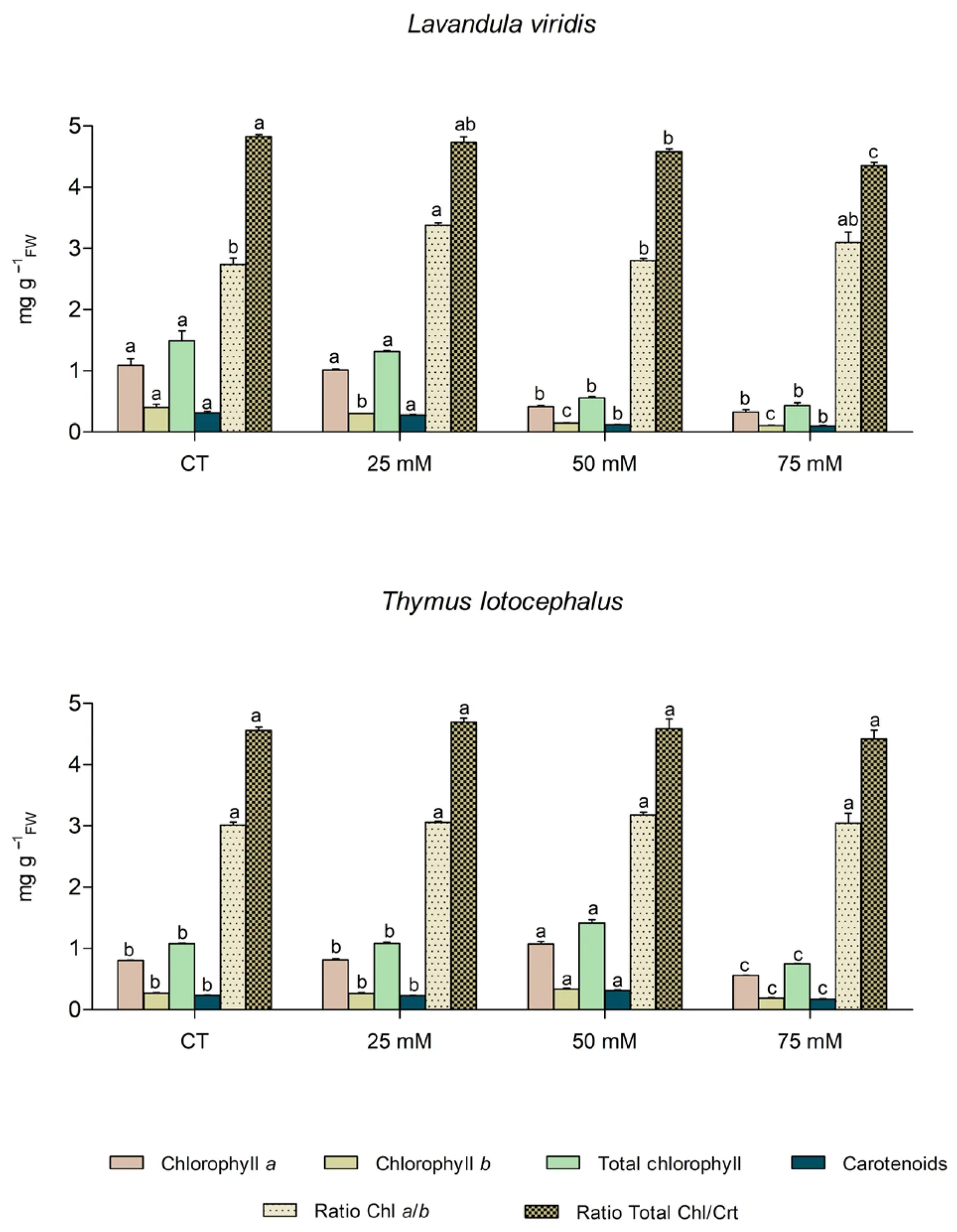
Chlorophyll *a*, chlorophyll *b*, total chlorophyll, and carotenoid contents, chlorophyll (Chl) *a/b* ratio, and total chlorophyll/carotenoid (Chl/Crt) ratio in *Lavandula viridis* and *Thymus lotocephalus* cultured on control medium (CT; 0 mM NaCl) and media with 25, 50, or 75 mM NaCl. Values are expressed as mean ± standard error (SE) and expressed as mg g^-1^ fresh weight (FW), except for Chl a/b and Chl/Crt ratios, which are dimensionless. Different letters indicate significant differences among NaCl treatments within each species according to Duncan’s multiple range test (*p* < 0.05).

Salinity impairs water and nutrient uptake, leading to ionic imbalance, osmotic stress, oxidative damage, and the disruption of essential metabolic processes. These pressures reduce cellular turgor and can damage the photosynthetic apparatus, ultimately inducing chlorosis and accelerating leaf senescence [32]. Figure 2 presents the effects of NaCl on the photosynthetic pigment contents and pigment ratios of *L. viridis* and *T. lotocephalus*. As chlorophyll and carotenoid contents are highly sensitive to environmental stress, they serve as reliable biochemical indicators of plant physiological status and salinity tolerance [33].

In *L. viridis*, chlorophyll *a*, total chlorophyll and carotenoid contents remained relatively stable at 25 mM NaCl but declined significantly at 50 and 75 mM. This salinity sensitivity is consistent with findings on *L. angustifolia*, where NaCl concentrations above 50 mM reduced chlorophyll *a* and *b* contents by up to 73% [21]. More recently, Miri et al. [28] demonstrated that pigment response is genotype-dependent, since *L. angustifolia* subsp. *angustifolia* exhibits greater pigment loss than subsp. *officinalis*. The progressive decline in chlorophyll *b* and in the total chlorophyll/carotenoid ratio observed in *L. viridis* suggests enhanced chlorophyll degradation under severe salinity, likely associated with chlorophyllase activation and thylakoid membrane disruption [28].

By contrast, *T. lotocephalus* maintained stable pigment levels at 25 mM NaCl and even showed increased chlorophyll and carotenoid contents at 50 mM. A marked reduction occurred only at 75 mM NaCl. This response diverges from the salt sensitivity reported for *T. vulgaris* and *T. daenensis*, where 60–90 mM NaCl significantly reduced chlorophyll *a*, chlorophyll *b*, and total chlorophyll contents [20]. Instead, *T. lotocephalus* behaves similarly to the salt-tolerant *T. pannonicus*, which maintains pigment stability under 60 mM NaCl [34]. The increase in photosynthetic pigments under moderate salinity may represent an adaptive strategy to maximize light harvesting and maintain the energetic requirements of stress defence mechanisms. Supporting this, Li et al. [35] found that moderate salinity upregulates photosystem II (PSII)-stabilizing genes (*LaPSB28*, *LaPSBS*, and *LaPSBR*) in lavender, preserving pigments and maintaining electron transport efficiency.

Previous studies showed that photosynthetic pigments of both species were also affected by other abiotic stresses. Under in vitro drought simulation conditions, 7% PEG reduced chlorophyll and carotenoid contents by more than 50% in *T. lotocephalus* [31]. Likewise, nitrogen and iron deficiencies caused reductions in pigment levels in both species, reflecting the fundamental roles of nitrogen as a structural component of chlorophyll molecules and iron as an essential cofactor in photosynthetic electron transport [12]. Thermal stress also significantly impaired pigment stability. Both low (15 °C) and high (30 °C) temperatures also reduced pigment contents, with heat stress causing the most severe declines (65–81%), probably as a consequence of thylakoid membrane disruption and protein degradation induced by excessive ROS production [15].

Pigment ratios further highlight the distinct responses of the two species to salinity under their respective optimized in vitro culture medium. Both the chlorophyll *a*/*b* and total chlorophyll/carotenoid ratios remained relatively stable across treatments in *T. lotocephalus*, indicating efficient pigment homeostasis and preservation of the photosynthetic apparatus (Figure 2). In contrast, *L. viridis* exhibited a progressive decrease in the total chlorophyll/carotenoid ratio, reaching its lowest value at 75 mM NaCl. This trend indicates faster degradation of chlorophyll than carotenoids, reflecting increasing oxidative stress and a greater dependence on carotenoid-mediated photoprotection to detoxify ROS [29]. The lowest value observed at 75 mM NaCl suggests that severe salinity compromises pigment homeostasis, likely by causing chloroplast damage and/or triggering a defensive adjustment, with carotenoids playing a relatively more important role in protecting against oxidative stress [10]. 

The chlorophyll *a*/*b* ratio provides additional insight into photosynthetic acclimation, as it reflects changes in PSII antenna size and the relative abundance of accessory pigments. An increase in this ratio generally indicates a loss of chlorophyll *b* and contraction of the light-harvesting antenna, which minimizes photodamage under stress conditions [24]. In *L. viridis*, the highest chlorophyll *a*/*b* ratio was recorded at 25 mM NaCl, suggesting an early acclimation response to mild salinity through antenna size adjustment before the onset of severe pigment degradation at higher salt concentrations. Although the ratio decreased at 50 mM NaCl, the relatively high values observed at 75 mM suggest that chlorophyll *b* remained more susceptible to salinity-induced degradation than chlorophyll *a*. This is consistent with progressive adjustments of the photosynthetic antenna complex under severe stress conditions. Comparable changes in chlorophyll/carotenoid and chlorophyll a/b ratios have been reported in *S. coccinea* [29] and *Lavandula officinalis* [36,37], reinforcing the role of these pigment ratios as sensitive indicators of salinity-induced stress in Lamiaceae species.

### 3.3. Oxidative Stress Markers

**Figure 3:**
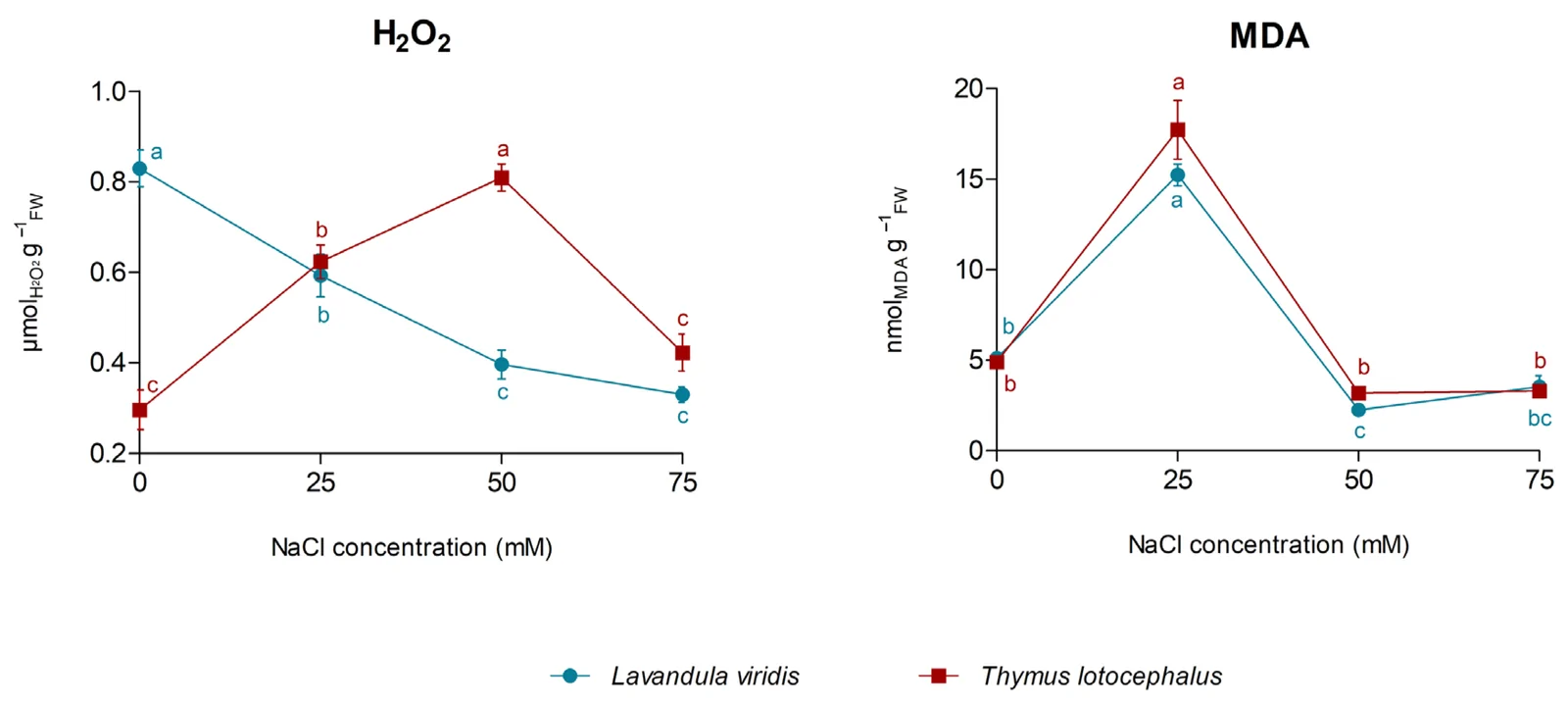
Hydrogen peroxide (H_2_O_2_) and malondialdehyde (MDA) contents in *Lavandula viridis* and *Thymus lotocephalus* cultured on control medium (CT; 0 mM NaCl) and media with 25, 50, or 75 mM NaCl. Values are presented as mean ± standard error (SE). Different letters indicate significant differences among NaCl treatments within each species according to Duncan’s multiple range test (*p* < 0.05).

To assess the salinity-induced oxidative stress and cellular damage in *L. viridis* and *T. lotocephalus*, H_2_O_2_, a major ROS, and MDA, a widely used marker of lipid peroxidation, were quantified under the different NaCl treatments. As shown in Figure 3, the two species exhibited opposing H_2_O_2_ accumulation patterns. 

In *L. viridis*, H_2_O_2_ levels progressively and significantly declined with increasing NaCl concentration, reaching reductions of approximately 52% and 60% (relative to the control) at 50 and 75 mM NaCl, respectively. This response diverges from several Lamiaceae species, such as *L. angustifolia* subsp. *officinalis* [28], *Dracocephalum kotschyi* [22] and *Rosmarinus officinalis* [38], which typically accumulate H_2_O_2_ under salinity. Conversely, *T. lotocephalus* showed a transient oxidative response, with H_2_O_2_ content increasing significantly at 25 mM NaCl and reaching a maximum at 50 mM (~174% above control), before declining to near-control levels at 75 mM NaCl. This contrasting pattern between the two species indicates a dual role of H_2_O_2_. While high levels are often associated with damage, the moderate increase observed in *T. lotocephalus* could indicate its capacity to act as a key signalling molecule rather than a purely toxic stress byproduct [39].

Lipid peroxidation patterns were similar in both species. Mild salinity (25 mM NaCl) induced pronounced MDA accumulation, reaching 15.24 nmol g^−1^_FW_ in *L. viridis* and 17.72 nmol g^−1^_FW_ in *T. lotocephalus*. These results indicate that the initial exposure to salinity caused substantial membrane damage and oxidative stress, reflecting the high sensitivity typically observed in glycophytes during the early stages of salt stress [10]. This transient MDA behaviour has also been reported in other Lamiaceae species, including *Mentha piperita* [40] and *Ocimum basilicum* [41]. Interestingly, at 50 and 75 mM NaCl, MDA levels returned to, or even fell below, those of the control.

The simultaneous decline in H_2_O_2_ and MDA observed at 75 mM NaCl suggests that both species underwent substantial metabolic adjustments in response to prolonged or severe salinity. Rather than signalling genuine stress tolerance, this pattern likely reflects a complex physiological acclimation involving diminished ROS generation paired with enhanced detoxification. At this salinity level, the marked decline in photosynthetic pigments (Figure 2) suggests compromised chloroplast function, which may have restricted photosynthetic electron transport and consequently limited ROS production. Simultaneously, non-enzymatic antioxidant systems, like carotenoids and phenolic compounds, may have contributed to ROS scavenging and the protection of cellular structures from oxidative damage [42,43]. This response is consistent with the growth–defence trade-off hypothesis, which proposes that, under severe environmental stress, plants reallocate metabolic resources from growth and primary metabolism towards protective and defence mechanisms [22]. This is supported by the substantial reductions in shoot growth and biomass observed at 75 mM NaCl (Figure 1 and Table 1), suggesting that resource allocation was shifted from growth to stress acclimation.

### 3.4. Osmotic Regulation

To investigate the osmotic adjustment mechanisms induced by salinity in *L. viridis* and *T. lotocephalus*, the accumulation of two major compatible solutes, proline and soluble sugars, was evaluated. Proline is one of the most effective osmoprotectants in plants, mitigating stress by acting as an intracellular osmolyte, ROS scavenger, molecular chaperone, and stabilizer of proteins and cellular membranes under dehydration conditions [10]. Soluble sugars also assist in osmotic balancing, acting both as compatible solutes and essential signalling molecules that regulate stress perception, carbon metabolism, and the maintenance of cellular homeostasis under abiotic constraints [24].

**Figure 4:**
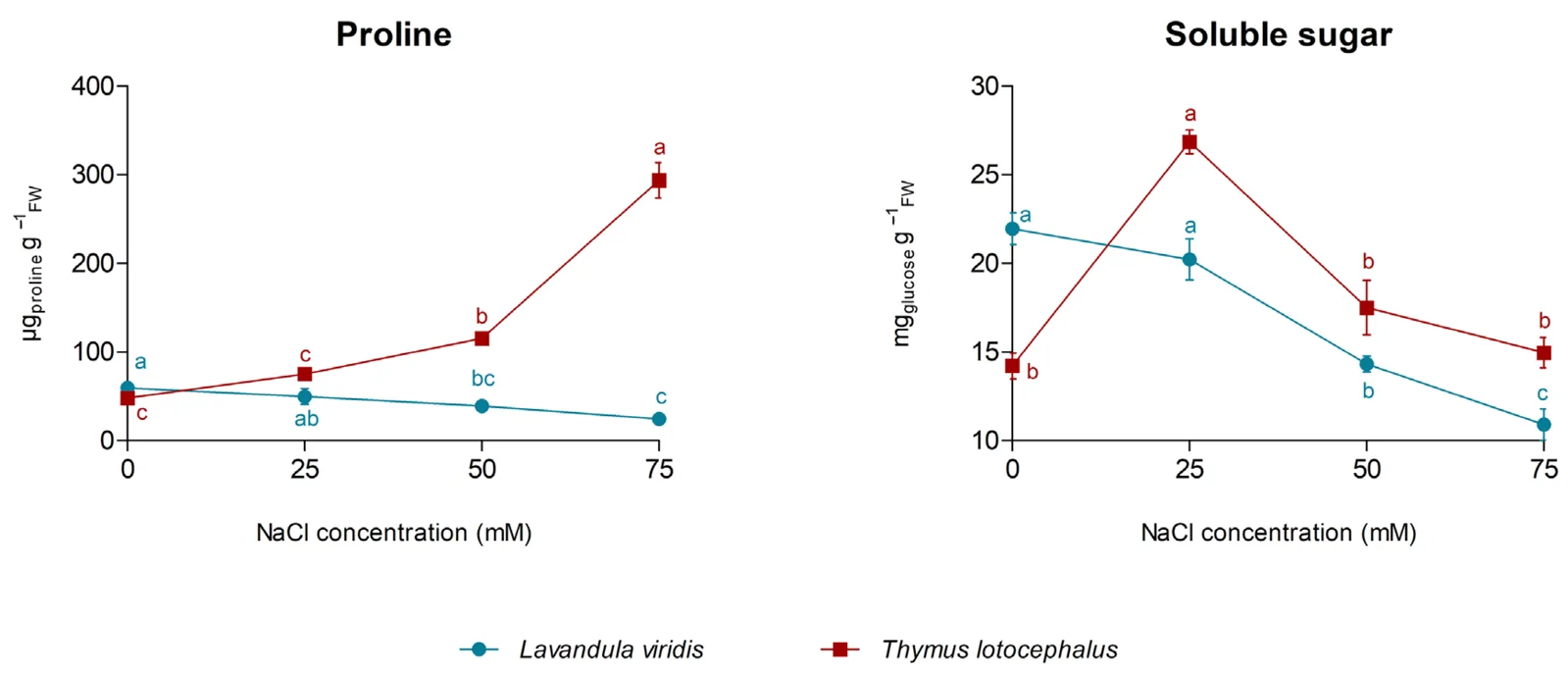
Proline and soluble sugar contents in *Lavandula viridis* and *Thymus lotocephalus* cultured on control medium (CT; 0 mM NaCl) and media supplemented with 25, 50, or 75 mM NaCl. Values are presented as mean ± standard error (SE). Different letters indicate significant differences among NaCl treatments within each species according to Duncan’s multiple range test (*p* < 0.05).

As shown in Figure 4, the two Lamiaceae species exhibited markedly different osmoregulatory responses to salinity. Proline content increased progressively up to 50 mM NaCl in *T. lotocephalus*, followed by a sharp rise at 75 mM, reaching 293.8 µg g^−1^_FW_ (~6-fold higher than the control). This pronounced accumulation is in accordance with previous studies on this species, in which proline also increased substantially under PEG-induced drought [31] and temperature stress (15 and 30 °C) [15]. In *T. vulgaris,* proline accumulation also constitutes an important component of the adaptive response to prolonged salinity [44]. Nevertheless, the magnitude of this response depends on stress duration. For example, *T. vulgaris* exposed to 100 mM NaCl showed an initial reduction in proline after two weeks, followed by a threefold increase after four weeks of treatment [39], highlighting the dynamic nature of proline metabolism during stress acclimation. In contrast, in *L. viridis*, proline levels decreased progressively as NaCl concentration increased, indicating a fundamentally different adaptive strategy. A similar decline has been described in *L. multifida*, where reduced proline levels were attributed to enhanced proline catabolism, providing energy and reducing power to sustain metabolic maintenance under severe salinity [30]. Although this mechanism was not investigated in the present study, it may partially explain the response observed in *L. viridis*. These behaviours contrast with that reported for *L. angustifolia*, which exhibits a dose-dependent accumulation of proline under salt stress [28]. 

The soluble sugar profile further emphasized the contrasting strategies between both species. In *L. viridis*, soluble sugars declined progressively with increasing salinity, indicating impaired osmotic adjustment. The simultaneous depletion of both proline and soluble sugars suggests that severe salinity compromised carbon assimilation and reduced the availability of compatible solutes required to maintain cellular osmotic balance. This response is consistent with the marked reduction in photosynthetic pigments (Figure 2) and growth (Figure 1 and Table 1), suggesting an overall metabolic limitation under high salinity. Consequently, the observed reduction in ROS and lipid peroxidation at the highest NaCl concentration is likely associated with alternative protective mechanisms rather than effective osmotic adjustment [10]. Conversely, *T. lotocephalus* accumulated soluble sugars under mild salinity (25 mM NaCl), a similar response to that previously observed under low-temperature stress (15 °C) [15]. At higher salinity levels (50 and 75 mM NaCl), however, soluble sugar concentrations declined to values close to those of the control, whereas proline accumulation increased markedly. This complementary behaviour suggests a coordinated osmotic response in which soluble sugars contribute to the initial acclimation to moderate stress, while proline becomes the predominant compatible solute under more severe salinity.

Overall, these findings indicate distinct osmoregulatory responses to salinity in the two species. Under their respective culture conditions, *T. lotocephalus* showed coordinated accumulation of compatible solutes, whereas *L. viridis* exhibited marked depletion of these compounds under increasing salinity. These observations suggest differences in their adaptive responses to salinity but should not be interpreted as direct quantitative comparisons between species. 

### 3.5. Regulation of Secondary Metabolism

#### 3.5.1. Phenolic Composition 

Salinity is a major abiotic stress that profoundly affects plant metabolism by triggering the reprogramming of secondary metabolic pathways as part of the adaptive defence response [6]. In the present study, *L. viridis* and *T. lotocephalus* exhibited divergent metabolic shifts to increasing concentrations of NaCl, highlighting species-specific strategies for coping with osmotic and ionic stress. Both phenolic profiles were dominated by phenolic acids, particularly RA, which accounted for 38% and 43% of the total phenolic content in control cultures of *L. viridis* and *T. lotocephalus*, respectively. This predominance confirms the central role of the RA biosynthetic pathway in the stress physiology of these Mediterranean Lamiaceae species. In total, 26 phenolic compounds were identified in *L. viridis* extracts, of which 20 were quantified, whereas 31 compounds were identified and 27 were quantified in *T. lotocephalus* (Table 2). Since phytochemical analyses were performed using extracts prepared from a fixed amount of dried plant material, the reported values reflect metabolite concentrations in the analyzed samples rather than total metabolite yields per culture. Therefore, the results should be interpreted as changes in metabolite accumulation patterns under salinity stress and not as estimates of overall metabolite productivity.

In *L. viridis*, the total phenolic content decreased progressively with increasing salinity (from 6710.82 µg g^−1^_DW_ in the control to 4637.13 µg g^−1^_DW_ at 75 mM NaCl). This response suggests that severe salinity progressively impaired secondary metabolism, a pattern previously reported in other Lamiaceae species, including *L. angustifolia* [21], *Lavandula coronopifolia* [45], *Salvia macrosiphon* [46], and *Thymus serpyllum* [47]. The reduction in phenolic accumulation is likely associated with the inhibition of carbon assimilation and photosynthetic performance observed under severe salinity, which limits the availability of ATP, reducing power, and carbon skeletons required for phenylpropanoid biosynthesis [22]. 

Despite the overall decline in total phenolics, phenolic acids remained the dominant metabolite class in *L. viridis*. RA was consistently the most abundant compound, although its concentration decreased from 3099.98 µg g^−1^_DW_ in the control to 2298.92 µg g^−1^_DW_ at 75 mM NaCl (Table 2). Responses of RA to salinity have been shown to be species- and genotype-dependent. For example, Scagel et al. [48] reported that salinity increased RA accumulation in *Ocimum basilicum* ‘Sweet Broadleaf’ but the opposite response occurred the ‘Siam Queen’ cultivar. A similar decline was observed for the major salvianolic acid isomers, particularly salvianolic acid B isomer III, whose concentration decreased ~43% under the highest salinity treatment. In contrast, some simple phenolic acids, notably ferulic acid, increased markedly, exhibiting a 181% increase at 75 mM NaCl, while caffeic acid showed a more modest rise. The accumulation of these precursor compounds may indicate the partial reorganization of phenylpropanoid metabolism, reflecting either reduced conversion into more complex derivatives or an increased requirement for these molecules as direct antioxidants under stress conditions [49].

Conversely, *T. lotocephalus* displayed a distinct metabolic response, accumulating phenolics under salinity. Total phenolic content increased by ~13% at 25 mM NaCl (5570.79 µg g^−1^_DW_), remained relatively stable at 50 mM, and declined only slightly at 75 mM NaCl (Table 2). This pattern indicates that mild and moderate salinity promoted the accumulation of phenolic compounds, whereas only severe stress approached the physiological tolerance threshold of the species.

RA remained the predominant phenolic compound in *T. lotocephalus*, but unlike *L. viridis*, its concentration increased under mild salinity, reaching 3193.34 µg g^−1^_DW_ at 25 mM NaCl and remaining high at 50 mM. Given the well-established antioxidant, anti-inflammatory, antimicrobial, and antitumoral properties of RA, this accumulation represents a relevant result [50]. Enhanced RA accumulation likely contributes to mitigating oxidative damage caused by salinity-induced ROS, reinforcing its central role in *T. lotocephalus*’s adaptive response. The salinity-induced stimulation of RA has also been reported in *T. vulgaris* and *T. daenensis*, where accumulation increased by up to 32% under saline conditions [20]. Likewise, *D. kotschyi* exhibited increased RA accumulation up to 75 mM NaCl, followed by a decline at higher salinity levels [22]. 

Beyond RA itself, *T. lotocephalus* showed a pronounced increase in several RA-derived metabolites. Total salvianolic acids increased by ~44%, from 923.28 µg g^−1^_DW_ in the control to 1324.75 µg g^−1^_DW_ at 75 mM NaCl (Table 2), largely driven by the strong accumulation of salvianolic acid B isomer III, whose concentration more than tripled under severe salinity. Similarly, yunnaneic acid F, salviaflaside isomers and methylrosmarinic acid isomer I increased by 55%, 50–62% and 75%, respectively, under mild salinity. Since these compounds arise from the oxidative coupling and structural diversification of RA [51], their increased accumulation under saline conditions is consistent with enhanced flux through RA-related phenolic metabolic pathways. This metabolic profile is also consistent with the growth–defence trade-off hypothesis, where carbon and energy may be preferentially allocated to secondary metabolism rather than biomass accumulation under stress conditions [22]. From a chemotaxonomic perspective, the predominance of RA and its derivatives in both species is noteworthy. These compounds are characteristic of the Lamiaceae family and are frequently associated with high antioxidant capacity [6,51]. Their abundance further supports the importance of phenolic acid metabolism in the response of these species to salinity-induced oxidative stress. 

Flavonoids, although recognized as effective antioxidants that complement the action of phenolic acids [52], showed minimal responses to salinity in both species. Luteolin-7-*O*-glucuronide was the predominant flavonoid in both species, although its concentration was substantially higher in *T. lotocephalus*. Its content progressively declined with increasing NaCl concentration, decreasing by ~25% at 75 mM. A similar pattern was observed for naringenin in *T. lotocephalus*. In *L. viridis*, total flavonoid content remained relatively stable throughout the treatments, suggesting that flavonoid accumulation was only marginally affected by salinity. The main exception was apigenin, whose concentration increased approximately fourfold at 50 mM NaCl (Table 2). 

The divergent metabolic responses observed between the two species possibly reflect differences in stress perception, phenylpropanoid pathway regulation, and metabolic plasticity [49]. Under the culture conditions used in this study, *T. lotocephalus* exhibited a coordinated accumulation of RA and its derivatives, together with increased H_2_O_2_ levels at 50 mM NaCl. The positive association between these responses is consistent with the involvement of redox-related signalling in the acclimation process, although the present data do not establish a causal relationship. In contrast, *L. viridis* showed progressive decreases in both H_2_O_2_ and phenolic compounds with increasing salinity, indicating a reduced capacity to maintain these responses under the experimental conditions. Whereas *T. lotocephalus* accumulated higher levels of RA and its derivatives under mild and moderate stress, *L. viridis* exhibited a progressive decline in these metabolites, which may be associated with reduced carbon availability and impaired photosynthetic performance under saline conditions [53]. These findings are fully consistent with the physiological responses described in the previous sections and highlight distinct acclimation patterns in the two species under their respective optimized culture conditions.

From an applied perspective, these results show that controlled salinity may represent a useful elicitation approach to enhance the accumulation of high-value phenolic compounds in *T. lotocephalus* cultures. A similar metabolic response was previously observed under iron deficiency, where RA accumulation increased by ~34% [12]. Likewise, under temperature stress, *T. lotocephalus* maintained a stable antioxidant system [15]. In contrast, osmotic stress induced by PEG reduced both growth and phenolic accumulation in *T. lotocephalus* [31].

#### 3.5.2. Antioxidant Activity of Extracts 

**Figure 5:**
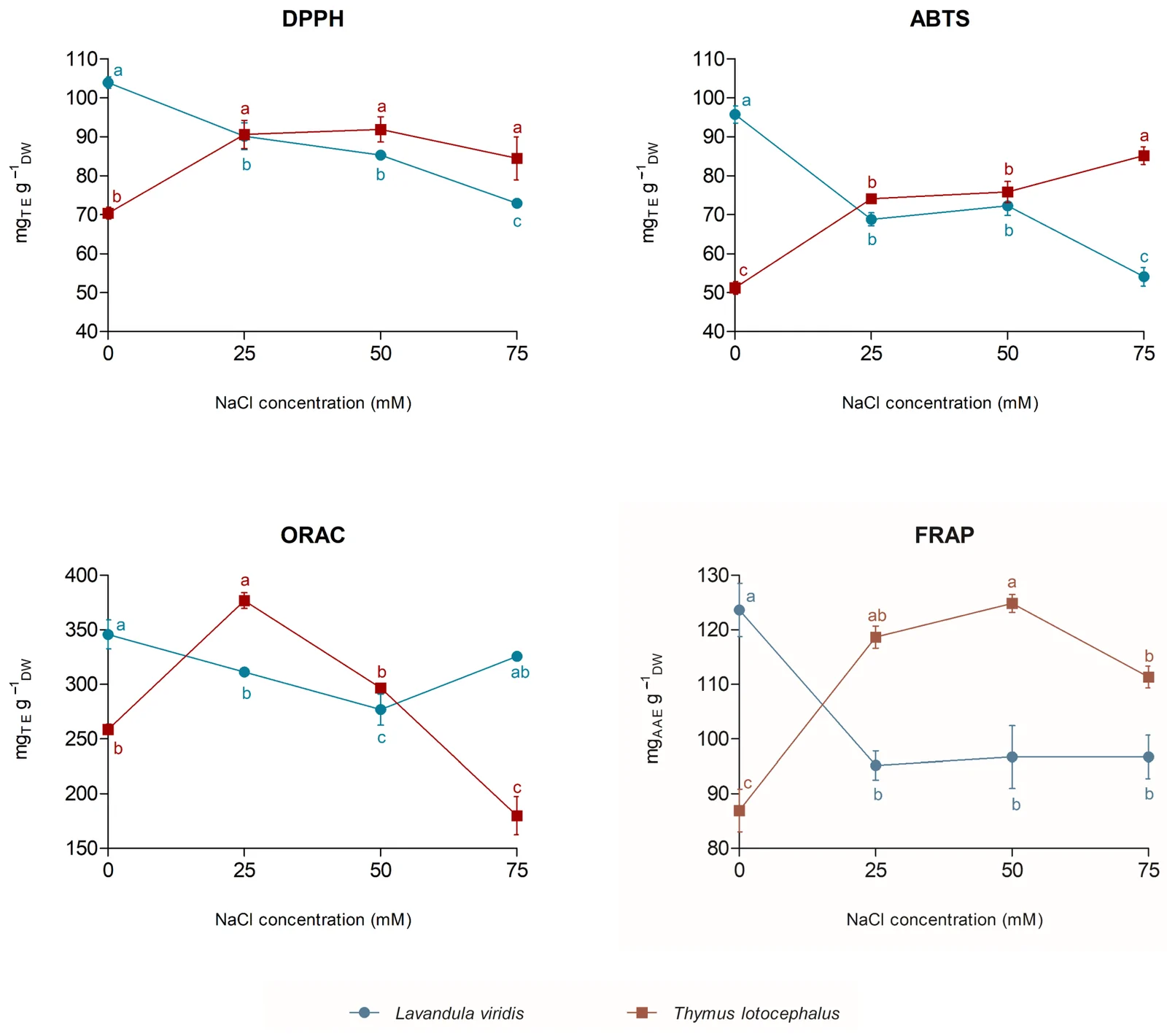
Antioxidant capacity determined by DPPH, ABTS, FRAP, and ORAC assays in *Lavandula viridis* and *Thymus lotocephalus* cultured on control medium (CT; 0 mM NaCl) and media with 25, 50, or 75 mM NaCl. Different letters indicate significant differences among NaCl treatments within each species according to Duncan’s multiple range test (*p* < 0.05).

The antioxidant capacity of *L. viridis* and *T. lotocephalus* extracts obtained from cultures grown under salinity stress closely reflected the contrasting metabolic responses described in the previous section (Figure 5). Overall, the antioxidant assays demonstrated that salinity progressively reduced the antioxidant capacity of *L. viridis* extracts, whereas *T. lotocephalus* extracts maintained or exhibited higher antioxidant capacity under mild to moderate salinity, highlighting contrasting responses to salt stress.

In *L. viridis*, DPPH and ABTS radical scavenging activities declined progressively with increasing NaCl concentration, indicating a gradual loss of antioxidant capacity. This reduction closely paralleled the decrease in total phenolic content, RA and salvianolic acid derivatives, suggesting that the lower antioxidant activity was associated with reduced accumulation of phenolic compounds. Similar results have been reported for *L. angustifolia*, where salinity levels above 50 mM significantly reduced antioxidant capacity, measured by DPPH, FRAP, and ABTS assays [21]. Likewise, the sharp reduction in FRAP values observed at 25 mM NaCl indicates that the reducing capacity of *L. viridis* was rapidly compromised under salt stress conditions.

In contrast, *T. lotocephalus* exhibited a distinct antioxidant response. DPPH and FRAP values increased under mild and moderate salinity (25 and 50 mM), while ABTS activity continued to increase throughout the salinity gradient, reaching its highest value at 75 mM NaCl. These results indicate that moderate salinity acted as an effective elicitor, being associated with higher extract antioxidant capacity and increased accumulation of phenolic compounds. This behaviour is in agreement with the enhanced accumulation of RA, salvianolic acids, and related phenolic derivatives observed under the same conditions and is consistent with reports for other salt-tolerant Lamiaceae species, including *T. vulgaris* and *T. daenensis*, in which antioxidant capacity increased under saline conditions [20]. The maintenance of the high antioxidant capacity of extracts of *T. lotocephalus* under mild to moderate salinity was accompanied by increased accumulation of phenolic compounds, including RA and salvianolic acid derivatives.

The ORAC assay exhibited a pattern distinct from the other antioxidant methods. At 75 mM NaCl, *L. viridis* showed partial recovery in ORAC values, whereas *T. lotocephalus* exhibited a marked decline. Since ORAC reflects hydrogen atom transfer (HAT) reactions, whereas FRAP is based on single-electron transfer (SET) and DPPH and ABTS involve both mechanisms, these contrasting responses likely indicate changes in the composition of the antioxidant pool rather than its overall abundance alone. In *L. viridis*, the partial recovery may indicate the relative contribution of specific hydrogen atom-donating compounds that remain active despite the overall reduction in phenolic content. Conversely, the decline in ORAC activity observed in *T. lotocephalus* at the highest salinity level suggests that the most severe treatment affected the contribution of some HAT-active antioxidants, despite the maintenance of high levels of phenolic compounds and strong antioxidant performance measured by the other assays.

Overall, the antioxidant results were consistent with the biochemical and metabolic patterns described throughout this study. *L. viridis* showed progressive metabolic impairment under salinity stress, characterized by reduced photosynthetic pigments, depletion of compatible solutes, the suppression of phenolic metabolism, and declining antioxidant capacity, whereas *T. lotocephalus* maintained coordinated biochemical and metabolic responses, including osmotic adjustment, increased accumulation of RA and its derivatives, and high antioxidant activity of the extract under the respective culture conditions. The association between phenolic accumulation and extract antioxidant capacity suggests an important contribution of phenolic acids, particularly RA and salvianolic derivatives, to the antioxidant properties of *T. lotocephalus* under salinity stress.

### 3.6. Multivariate Analysis

**Figure 6:**
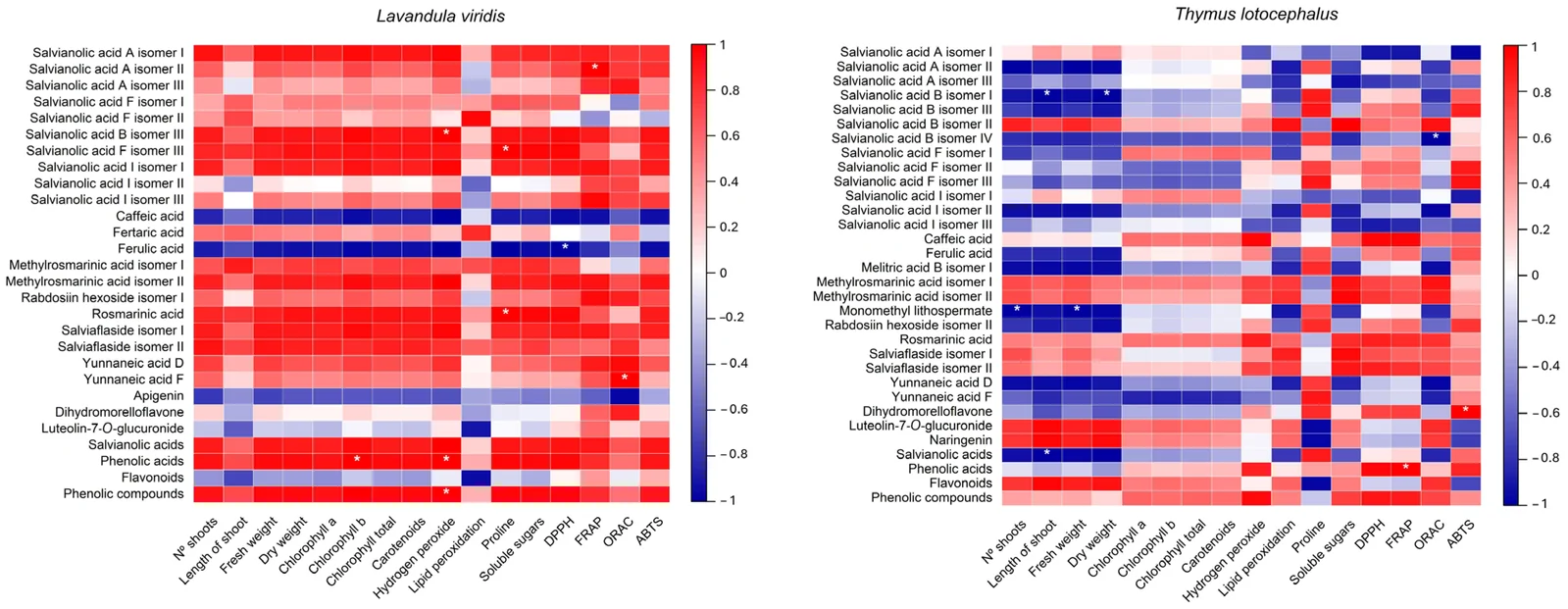
A heatmap showing the Pearson correlation coefficients between growth parameters, photosynthetic pigments, oxidative stress markers, osmolytes, antioxidant capacity, and individual and grouped phenolic compounds in *Lavandula viridis* and *Thymus lotocephalus* cultured on control medium (CT; 0 mM NaCl) and media with 25, 50, or 75 mM NaCl. Positive correlations are shown in red and negative correlations in blue, with colour intensity proportional to the correlation coefficient (−1 to +1). Asterisks (*) indicate statistically significant correlations (*p* ≤ 0.01). Phenolic compounds are identified according to their UHPLC–HRMS-MS characterization, and grouped variables include total salvianolic acids, total flavonoids, total phenolic acids, and total phenolic compounds.

**Figure 7:**
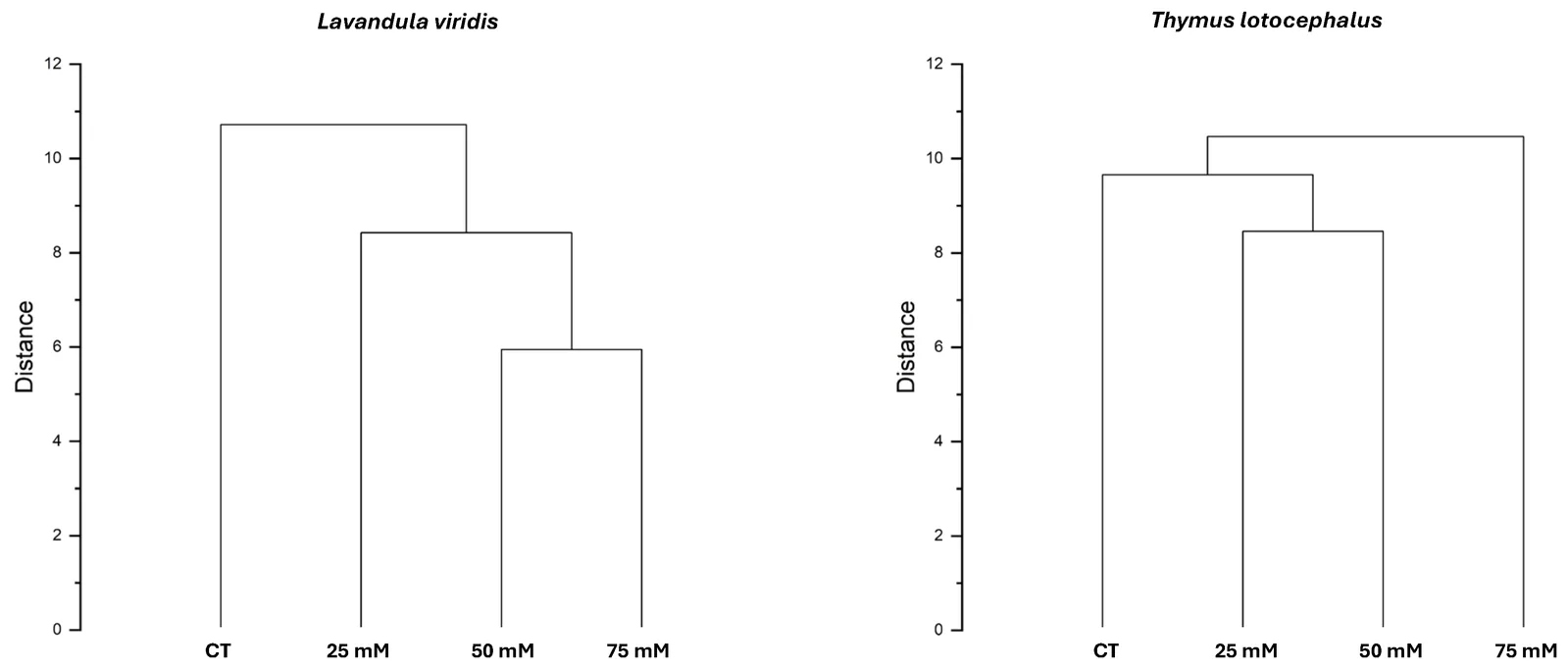
Dendrograms of the hierarchical cluster analysis (HCA) based on Euclidean distance for *Lavandula viridis* and *Thymus lotocephalus* cultured on control medium (CT; 0 mM NaCl) and media with 25, 50, or 75 mM NaCl. The vertical axis represents the linkage distance between clusters.

Multivariate analyses including the heatmap (Figure 6) and hierarchical cluster analysis (Figure 7), integrating all morphological, biochemical, and metabolic variables, further highlighted the distinct response patterns displayed by *L. viridis* and *T. lotocephalus* in response to salinity.

In *T. lotocephalus,* the heatmap revealed a more heterogeneous correlation network, reflecting its greater metabolic plasticity. RA and several related metabolites, including salviaflaside and methylrosmarinic acid isomers, were positively associated with antioxidant activity, whereas other compounds showed negative correlations with growth- and pigment-related variables. This pattern suggests that different groups of phenolic compounds exhibited distinct responses to increasing salinity. The negative associations observed for salvianolic acids, ferulic acid, monomethyl lithospermate, rabdosiin hexoside isomer I, and yunnaneic acids D and F are consistent with a metabolic reallocation from primary growth towards the synthesis of protective secondary metabolites under saline conditions. In contrast, flavonoids were positively correlated with growth-related traits, indicating that their accumulation was associated with the maintenance of physiological performance rather than representing an additional metabolic cost. Following z-score standardization, hierarchical cluster analysis revealed that the 25 and 50 mM NaCl treatments were the most similar, clustering together at the shortest distance. This cluster subsequently grouped with the control treatment, whereas the 75 mM NaCl treatment remained separated until the final clustering stage, indicating a markedly different response under severe salinity conditions. The proximity between the 25 and 50 mM treatments suggests the occurrence of a common metabolic adjustment to mild and moderate salt stress, reflected in coordinated changes in growth, biochemical parameters, and antioxidant responses. In contrast, the separation of the 75 mM treatment indicates that severe salinity induced a broader shift in the physiological and metabolic profile of *T. lotocephalus*, reflecting a more pronounced stress response. 

In *L. viridis*, the heatmap revealed a relatively coordinated correlation pattern between RA, its derivatives, and antioxidant capacity, indicating that phenolic acids were closely associated with the extract’s antioxidant responses observed under salt stress (Figure 6). Most phenolic compounds exhibited positive correlations with growth- and pigment-related parameters, suggesting that higher levels of phenolic compounds were associated with improved physiological performance. Conversely, the decline in these metabolites at higher salinity levels coincided with reduced biomass production, pigment degradation, and lower antioxidant activity, reinforcing the overall metabolic impairment previously described. A contrasting clustering pattern was observed in *L. viridis*. The 50 and 75 mM NaCl treatments clustered together at the shortest distance, indicating a high degree of similarity in their overall physiological and metabolic responses under moderate to severe salinity. In contrast, the control and 25 mM NaCl treatments were more distinct and joined the cluster at later stages. The close association between the 50 and 75 mM treatments suggests that salinity levels above 25 mM induced marked changes in growth, biochemical, and metabolic parameters. Furthermore, the clear separation of these treatments from the control indicates that *L. viridis* responded differently once a critical salinity threshold was exceeded. This is in agreement with the reductions in growth and photosynthetic performance observed throughout the study.

Overall, the multivariate analyses corroborate the morphological, biochemical, and metabolic findings, revealing distinct response patterns to salinity in the two species. *T. lotocephalus* exhibited coordinated changes in metabolite accumulation, antioxidant capacity, and growth-related parameters across the salinity treatments, whereas *L. viridis* showed a progressive decline in several physiological and biochemical parameters with increasing salinity.

## 4. Conclusions

This study demonstrates that *T. lotocephalus* and *L. viridis* exhibit markedly divergent physiological and metabolic responses to salinity under their respective optimized in vitro culture conditions. The results indicate that the ability to maintain redox homeostasis was associated with different acclimation patterns in the two species. Under the experimental conditions tested, *T. lotocephalus* displayed a great metabolic plasticity, characterized by efficient osmotic adjustment, enhanced accumulation of proline, RA, salvianolic acids, and related phenolic derivatives, and sustained antioxidant capacity under mild to moderate salinity. In contrast, *L. viridis* showed progressive reductions in growth, photosynthetic pigments, phenolic metabolism, and antioxidant activity as salinity increased, indicating a comparatively lower tolerance to NaCl concentrations above 25 mM.

RA and its derivatives emerged as key metabolites associated with salinity tolerance, showing strong relationships with antioxidant capacity and supporting their central role in protecting plants against oxidative stress. Multivariate analyses further highlighted distinct metabolic response patterns in the two species, with coordinated metabolic adjustments in *T. lotocephalus* and progressive metabolic impairment in *L. viridis*.

Overall, this work provides new insights into the biochemical and metabolic responses of two Mediterranean endemic Lamiaceae species to salinity. From a biotechnological perspective, these findings demonstrate that controlled salinity can be successfully exploited as an elicitation strategy for enhancing the accumulation of valuable phenolic compounds with antioxidant capacity in *T. lotocephalus* in vitro cultures. This approach offers a sustainable and environmentally friendly alternative for increasing the production of bioactive metabolites of pharmaceutical, nutraceutical, cosmetic, and food interest, while contributing to the conservation and valorization of Mediterranean endemic plant species.

## Figures and Tables

**Table 1:** Shoot growth parameters of *Lavandula viridis* and *Thymus lotocephalus* cultured on control medium (CT; 0 mM NaCl) and media with 25, 50, or 75 mM NaCl.

Treatment	No. Shoots	Length of Longest Shoot (mm)	Fresh Weight (g)	Dry Weight (g)
*L. viridis*				
CT	5.60 ± 0.67 ^a^	30.55 ± 2.61 ^b^	1.38 ± 0.13 ^a^	0.11 ± 0.01 ^a^
25 mM	5.13 ± 0.54 ^a^	36.63 ± 2.06 ^a^	1.19 ± 0.13 ^a^	0.10 ± 0.01 ^a^
50 mM	2.92 ± 0.17 ^b^	23.58 ± 1.58 ^c^	0.49 ± 0.05 ^b^	0.07 ± 0.01 ^b^
75 mM	3.13 ± 0.25 ^b^	20.63 ± 1.33 ^c^	0.50 ± 0.05 ^b^	0.06 ± 0.00 ^b^
*T. lotocephalus*				
CT	7.71 ± 1.78 ^b^	29.92 ± 2.89 ^ab^	0.75 ± 0.06 ^b^	0.09 ± 0.00 ^a^
25 mM	12.08 ± 0.99 ^a^	33.79 ± 2.56 ^a^	1.08 ± 0.16 ^a^	0.10 ± 0.01 ^a^
50 mM	5.50 ± 0.75 ^b^	24.96 ± 0.80 ^b^	0.53 ± 0.08 ^bc^	0.07 ± 0.01 ^b^
75 mM	4.59 ± 1.22 ^b^	17.86 ± 2.25 ^c^	0.38 ± 0.09 ^c^	0.05 ± 0.01 ^b^

Values are presented as the mean ± SE. For each species and variable, differences among NaCl treatments were analyzed by one-way analysis of variance (ANOVA), followed by Duncan’s multiple range test. Means followed by different letters (a–c) within the same species indicate significant differences among treatments (*p* < 0.05).

**Table 2:** Qualitative and quantitative (µg g ^−1^_DW_, mean ± SE) analysis by UHPLC-HRMS-MS of the phenolic profile of extracts from *Lavandula viridis* and *Thymus lotocephalus* cultured on control medium (CT; 0 mM NaCl) and media with 25, 50, or 75 mM NaCl.

Compound	*Lavandula viridis*				*Thymus lotocephalus*			
	CT	25	50	75	CT	25	50	75
**Phenolic acids**								
Salvianolic acid A isomer I	7.14 ± 0.24 a	6.41 ± 0.19 b	5.71 ± 0.01 c	5.93 ± 0.07 bc	6.00 ± 0.05 a	5.84 ± 0.14 a	5.85 ± 0.16 a	5.83 ± 0.15 a
Salvianolic acid A isomer II	39.75 ± 0.93 a	25.18 ± 0.01 b	25.03 ± 0.93 b	27.25 ± 0.99 b	88.28 ± 2.91 b	58.13 ± 2.50 c	125.05 ± 8.63 a	129.08 ± 5.97 a
Salvianolic acid A isomer III	10.53 ± 0.13 a	8.45 ± 0.01 c	8.22 ± 0.11 c	9.63 ± 0.10 b	63.15 ± 6.61 a	40.77 ± 1.82 c	54.23 ± 5.42 ab	53.62 ± 0.81 ab
Salvianolic acid B isomer I	n.d	n.d	n.d	n.d	8.07 ± 0.55 bc	7.26 ± 0.49 c	9.94 ± 0.68 ab	11.45 ± 0.74 a
Salvianolic acid B isomer II	n.d	n.d	n.d	n.d	47.99 ± 4.52 b	62.08 ± 2.30 a	51.86 ± 0.65 b	46.58 ± 0.84 b
Salvianolic acid B isomer III	2485.00 ± 31.87 a	1857.25 ± 21.23 b	1540.37 ± 46.11 c	1405.67 ± 19.25 d	127.89 ± 2.23 d	181.58 ± 3.94 c	312.17 ± 11.60 b	392.64 ± 1.81 a
Salvianolic acid B isomer IV	n.d	n.d	n.d	n.d	92.36 ± 1.20 b	71.53 ± 5.20 c	83.41 ± 2.16 bc	108.02 ± 5.52 a
Salvianolic acid F isomer I	13.21 ± 3.19 a	14.17 ± 0.73 a	14.23 ± 0.28 a	10.38 ± 0.82 a	12.40 ± 0.77 a	11.94 ± 0.15 a	13.75 ± 0.22 a	12.75 ± 0.77 a
Salvianolic acid F isomer II	21.41 ± 0.75 c	56.24 ± 0.36 a	18.09 ± 0.20 d	28.20 ± 0.74 b	13.14 ± 0.28 a	39.98 ± 1.61 a	25.61 ± 1.08 a	47.72 ± 0.39 a
Salvianolic acid F isomer III	13.29 ± 0.13 a	13.01 ± 0.49 a	12.63 ± 0.49 a	11.96 ± 0.00 a	7.18 ± 0.18 c	8.51 ± 0.12 b	8.23 ± 0.05 b	9.86 ± 0.32 a
Salvianolic acid I isomer I	170.62 ± 6.78 a	78.14 ± 2.09 b	37.97 ± 3.25 c	36.99 ± 2.00 d	33.97 ± 1.26 a	30.80 ± 1.45 a	32.43 ± 1.24 a	30.54 ± 0.25 a
Salvianolic acid I isomer II	159.12 ± 4.81 a	102.19 ± 9.34 c	116.68 ± 13.02 bc	146.87 ± 4.47 ab	300.07 ± 7.42 b	201.59 ± 9.44 c	302.05 ± 5.76 b	381.42 ± 5.13 a
Salvianolic acid I isomer III	30.94 ± 1.02 a	19.28 ± 1.35 b	21.00 ± 1.59 b	22.48 ± 0.97 b	122.80 ± 0.12 a	70.68 ± 1.10 c	92.56 ± 2.44 b	95.22 ± 3.03 b
**Total salvianolic acids**	2951.01 ± 41.70 a	2180.33 ± 29.93 b	1799.92 ± 65.01 c	1705.36 ± 27.78 c	923.28 ± 25.28 c	790.70 ± 27.81 d	1117.14 ± 39.65 b	1324.75 ± 15.50 a
Caffeic acid	57.83 ± 1.10 b	64.76 ± 2.76 ab	67.05 ± 2.09 a	68.44 ± 2.09 a	40.78 ± 1.55 c	65.10 ± 2.42 a	70.20 ± 1.43 a	52.35 ± 0.77 b
Fertaric acid	145.32 ± 1.27 b	187.31 ± 1.35 a	105.43 ± 3.50 c	157.28 ± 5.95 b	<LOQ	<LOQ	<LOQ	<LOQ
Ferulic acid	40.34 ± 1.11 d	72.06 ± 0.47 c	90.63 ± 2.19 b	113.54 ± 2.93 a	<LOQ	<LOQ	2.14 ± 0.49	1.82 ± 0.59
Melitric acid B isomer I	<LOQ	<LOQ	<LOQ	<LOQ	10.91 ± 0.35 b	8.68 ± 0.32 c	11.66 ± 0.69 ab	13.72 ± 0.71 a
Methylrosmarinic acid isomer I	118.03 ± 1.87 b	139.47 ± 5.06 a	112.17 ± 6.82 b	69.09 ± 1.59 c	26.32 ± 0.84 c	46.16 ± 0.45 a	38.13 ± 0.02 b	25.57 ± 0.78 c
Methylrosmarinic acid isomer II	26.01 ± 4.35 a	19.74 ± 3.67 a	17.00 ± 2.54 a	16.36 ± 2.82 a	15.81 ± 2.47 a	26.88 ± 5.83 a	21.71 ± 4.03 a	17.40 ± 3.25 a
Monomethyl lithospermate	n.d	n.d	n.d	n.d	9.37 ± 1.10 a	8.16 ± 0.62 a	10.30 ± 1.07 a	10.66 ± 1.42 a
Rabdosiin hexoside isomer I	19.95 ± 0.01 a	13.06 ± 0.14 b	12.42 ± 0.22 c	14.83 ± 0.44 c	n.d	n.d	n.d	n.d
Rabdosiin hexoside isomer II	n.d	n.d	n.d	n.d	6.52 ± 0.13 b	6.64 ± 0.08 b	7.36 ± 0.27 a	7.50 ± 0.13 a
Rosmarinic acid	3099.98 ± 135.37 a	2903.25 ± 52.57 ab	2685.12 ± 81.36 b	2298.92 ± 63.51 c	2529.24 ± 55.14 b	3193.34 ± 85.19 a	3079.33 ± 104.76 a	2645.89 ± 16.59 b
Salviaflaside isomer I	62.23 ± 0.03 a	44.10 ± 0.27 b	33.33 ± 0.00 c	34.83 ±1.18 c	27.34 ± 1.37 c	40.97 ± 1.04 a	31.45 ± 0.22 b	32.43 ± 0.83 b
Salviaflaside isomer II	84.40 ± 2.42 a	80.83 ± 1.55 a	51.77 ± 3.78 c	66.52 ± 3.34 b	59.52 ± 1.95 c	96.36 ± 3.92 a	81.49 ± 1.37 b	72.79 ± 2.17 b
Yunnaneic acid D	18.66 ± 0.58 a	13.12 ± 0.06 b	10.22 ± 0.03 c	13.71 ± 0.34 b	19.24 ± 0.45 ab	17.62 ± 0.70 b	19.40 ± 0.15 ab	20.71 ± 0.84 a
Yunnaneic acid F	35.45 ± 0.14 a	26.40 ± 0.47 c	17.84 ± 1.59 d	31.10 ± 1.19 b	57.84 ± 2.39 b	50.09 ± 1.76 c	50.36 ± 0.30 c	89.76 ± 2.20 a
**Total phenolic acids**	6659.23 ± 91.57 a	5744.43 ± 29.33 b	5002.91 ± 15.79 c	4589.97 ± 94.18 d	3726.16 ± 85.96 b	4350.69 ± 116.05 a	4540.67 ± 144.27 a	4315.33 ± 0.65 a
**Flavonoids**								
Apigenin	2.26 ± 0.04 c	4.36 ± 0.36 b	9.33 ± 0.53 a	4.48 ± 0.24 b	<LOQ	<LOQ	<LOQ	<LOQ
Dihydromorelloflavone	5.29 ± 0.03 a	4.07 ± 0.29 b	3.94 ± 0.11 b	5.27 ± 0.12 a	2.58 ± 0.01 a	2.91 ± 0.25 a	2.96 ± 0.16 a	3.15 ± 0.14 a
Luteolin	<LOQ	<LOQ	<LOQ	<LOQ	<LOQ	<LOQ	<LOQ	<LOQ
Luteolin-7-*O*-glucuronide	44.04 ± 0.61 a	22.46 ± 0.98 b	40.64 ± 4.03 a	37.41 ± 5.13 a	1018.71 ± 2.26 a	1045.19 ± 11.66 a	938.14 ± 17.02 b	762.69 ± 19.81 c
Naringenin	<LOQ	<LOQ	<LOQ	<LOQ	171.66 ± 6.01 a	171.99 ± 5.43 a	93.88 ± 26.28 b	<LOQ
**Total flavonoids**	51.59 ± 0.68 a	30.90 ± 0.91 b	53.91 ± 3.61 a	47.15 ± 4.77 a	1192.94 ± 3.76 a	1220.10 ± 16.84 a	1034.98 ± 9.42 b	765.84 ± 19.67 c
**Total phenolic compounds**	6710.82 ± 92.25 a	5775.33 ± 28.42 b	5056.82 ± 12.18 c	4637.13 ± 89.41 d	4919.11 ± 89.72 b	5570.79 ± 132.90 a	5575.65 ± 134.85 a	5081.17 ± 20.32 b

Notes: n.d.: not detected; LOD: limit of detection; LOQ: limit of quantification. The results were analyzed using a one-way analysis of variance (ANOVA) followed by Duncan’s new multiple range test. For each species. different letters (a–d) in each row indicate significant differences (*ρ* < 0.05) among treatments.

## Data Availability

The raw data supporting the conclusions of this article will be made available by the authors on request.

## Supplementary Materials

The following supporting information can be downloaded at https://www.mdpi.com/article/10.3390/biom16081178/s1: Table S1. HPLC-HRMS data of identified phenolic compounds in *Lavandula viridis* and *Thymus lotocephalus* extracts; Table S2. Summary of HPLC-HRMS criterion for quantification of phenolic compounds in *Lavandula viridis* and *Thymus lotocephalus* extracts.

## Abbreviations

The following abbreviations are used in this manuscript:

NaCl	Sodium chloride
H_2_O_2_	Hydrogen peroxide
MDA	Malondialdehyde
ROS	Reactive Oxygen Species
NADES	Natural Deep Eutectic Solvent
UAE	Ultrasound-Assisted Extraction
RA	Rosmarinic acid
UHPLC-HRMS-MS	Ultra-High-Performance Liquid Chromatography coupled with High-Resolution Mass Spectrometry
