# Supplementary material for: Salinity-Induced Modulation of Phenolic Compounds and Antioxidant Activity in Lavandula viridis and Thymus lotocephalus
Source: Biomolecules. 2026 Aug 12;16(8):1178. doi: 10.3390/biom16081178 (PMC13511616; doi:10.3390/biom16081178)
Supplement: Supplementary file 1 [file biomolecules-16-01178-s001.zip › biomolecules-4460889-supplementary.pdf]

Supplementary information

Table S1. HPLC-HRMS data of identified phenolic compounds in *Lavandula viridis* and *Thymus lotocephalus* extracts.

Compound identity	Chemical formula	Theoretical exact mass $[M-H]^-$ *(m/z)*	Delta ppm (error)	RT (min)	MSI MI level*
Caffeic acid	$C_9H_8O_4$	179.0349	6.059	7.16	1
Fertaric acid [2]	$C_{14}H_{14}O_9$	325.0565	3.789	7.14	2
Salvianolic acid B isomer I [1]	$C_{36}H_{30}O_{16}$	717.1461	3.164	7.96	2
Salvianolic acid A isomer I [1]	$C_{26}H_{22}O_{10}$	493.114	0.784	8.07	2
Salvianolic acid I isomer I [1]	$C_{27}H_{22}O_{12}$	537.1038	1.764	8.34	2
Yunnaneic acid F [1]	$C_{29}H_{26}O_{14}$	597.1249	1.772	8.38	2
Yunnaneic acid D [1]	$C_{27}H_{24}O_{12}$	539.1194	2.499	8.48	2
Salviaflaside isomer I [1]	$C_{24}H_{26}O_{13}$	521.13	2.347	8.55	2
Dihydromorelloflavone [3]	$C_{30}H_{22}O_{11}$	557.1089	-1.396	8.56	2
Luteolin-7-*O*-glucuronide	$C_{21}H_{18}O_{12}$	461.0725	2.099	8.69	1
Ferulic acid	$C_{10}H_{10}O_4$	193.0506	5.204	8.71	1
Salvianolic acid B isomer II [1]	$C_{36}H_{30}O_{16}$	717.1461	1.881	9.06	2
Rabdosiin hexoside isomer I [1]	$C_{42}H_{40}O_{21}$	879.1989	1.849	9.13	2
Salvianolic acid A isomer II [1]	$C_{26}H_{22}O_{10}$	493.114	2.407	9.62	2
Rosmarinic acid	$C_{18}H_{16}O_8$	359.0772	1.939	9.62	1
Salviaflaside isomer II [1]	$C_{24}H_{26}O_{13}$	521.13	1.982	9.64	2
Salvianolic acid F isomer I [1]	$C_{17}H_{14}O_6$	313.0717	4.042	9.71	2
Salvianolic acid I isomer II [1]	$C_{27}H_{22}O_{12}$	537.1038	1.988	9.95	2
Salvianolic acid B isomer III [1]	$C_{36}H_{30}O_{16}$	717.1461	1.114	10.06	2
Rabdosiin hexoside isomer II [1]	$C_{42}H_{40}O_{21}$	879.1989	2.338	10.42	2
Salvianolic acid I isomer III [1]	$C_{27}H_{22}O_{12}$	537.1038	2.099	10.43	2
Salvianolic acid A isomer III [1]	$C_{26}H_{22}O_{10}$	493.114	2.326	10.47	2
Methylrosmarinic acid isomer I [1]	$C_{19}H_{18}O_8$	373.0929	2.375	10.53	2
Luteolin	$C_{15}H_{10}O_6$	285.0404	3.984	10.58	1
Salvianolic acid B isomer IV [1]	$C_{36}H_{30}O_{16}$	717.1461	1.965	11.12	2
Monomethyl lithospermate [1]	$C_{28}H_{24}O_{12}$	551.1195	1.774	11.17	2
Methylrosmarinic acid isomer II [1]	$C_{19}H_{18}O_8$	373.0929	2.214	11.39	2
Naringenin	$C_{15}H_{12}O_5$	271.0611	2.214	11.4	1
Apigenin	$C_{15}H_{10}O_5$	269.0455	3.792	11.79	1
Salvianolic acid F isomer II [1]	$C_{17}H_{14}O_6$	313.0717	3.563	11.95	2
Melitric acid B isomer I [1]	$C_{27}H_{20}O_{11}$	519.0932	2.066	12.87	2
Salvianolic acid F isomer III [1]	$C_{17}H_{14}O_6$	313.0717	2.668	13.16	2

*Metabolite standards initiative metabolite identification (MSI MI) levels. Reference standards were available for all compounds identified at MSI MI level 1. Results expressed as equivalents of rosmarinic acid (1), caffeic acid (2), quercetin (3)

Table S2. Summary of HPLC-HRMS criterion for quantification of phenolic compounds in *Lavandula viridis* and *Thymus lotocephalus* extracts.

Compound	Linear range (mg/L)	Intercept	Slope	R^2	LOD (µg/L)	LOQ (µg/L)
Rosmarinic acid	0.14-35	243285019.3	21051013.11	0.9987	0.11	0.34
Caffeic acid	0.02-1.41	4843780.715	238346539.2	0.9981	0.23	0.71
Quercetin	0.02-0.63	-6429021.894	367573950	0.9991	0.06	0.17
Luteolin	0.04-1.17	24279610.59	543764431.7	0.999	0.02	0.06
Luteolin-7-O-glucuronide	0.55-17.5	49107875.08	84569783.36	0.9992	0.45	1.38
Ferulic acid	0.01-1.17	-91074.52931	65987161.05	0.9997	0.75	2.26
Naringenin	0.08-40	-57899.45082	1003404.918	0.999	49.61	150.35
Apigenin	0.02-0.7	15621389.24	951358540.1	0.9992	0.02	0.08
